# Retinoic Acid Specifically Enhances Embryonic Stem Cell Metastate Marked by Zscan4

**DOI:** 10.1371/journal.pone.0147683

**Published:** 2016-02-03

**Authors:** Daniela Tagliaferri, Maria Teresa De Angelis, Nicola Antonino Russo, Maria Marotta, Michele Ceccarelli, Luigi Del Vecchio, Mario De Felice, Geppino Falco

**Affiliations:** 1 Biogem, Istituto di Ricerche Genetiche Gaetano Salvatore Biogem scarl, Ariano Irpino, Italy; 2 Qatar Computing Research Institute, HBKU, Doha, Qatar; 3 Department of Molecular Medicine and Medical Biotechnologies, University of Naples “Federico II”, Naples, Italy; 4 Department of Science, Università degli Studi del Sannio, Benevento, Italy; 5 Department of Biology, University of Naples “Federico II”, Naples, Italy; Laboratoire de Biologie du Développement de Villefranche-sur-Mer, FRANCE

## Abstract

Pluripotency confers Embryonic Stem Cells (ESCs) the ability to differentiate in ectoderm, endoderm, and mesoderm derivatives, producing the majority of cell types. Although the majority of ESCs divide without losing pluripotency, it has become evident that ESCs culture consists of multiple cell populations with different degrees of potency that are spontaneously induced in regular ESC culture conditions. *Zscan4*, a key pluripotency factor, marks ESC subpopulation that is referred to as high-level of pluripotency metastate. Here, we report that in ESC cultures treated with retinoic acid (RA), Zscan4 ESCs metastate is strongly enhanced. In particular, we found that induction of Zscan4 metastate is mediated *via* RA receptors (RAR-*alpha*, RAR-*beta*, and RAR-*gamma*), and it is dependent on phosphoinositide-3-kinase (PI3K) signaling. Remarkably, Zscan4 metastate induced by RA lacks canonical pluripotency genes *Oct3/4* and *Nanog* but retained both self-renewal and pluripotency capabilities. Finally we demonstrated that the conditional ablation of Zscan4 subpopulation is dispensable for both endoderm and mesoderm but is required for ectoderm lineage. In conclusion, our research provides new insights about the role of RA signaling during ESCs high pluripotency metastate fluctuation.

## Introduction

Embryonic stem cells (ESCs) are derived from the inner cell mass (ICM) of blastocyst and are characterized by two main peculiarities, namely self-renewal and pluripotency: self-renewal is defined as the symmetrical division of ESCs into identical undifferentiated daughter cells; pluripotency confers ESCs the ability to produce the majority of cell types upon appropriate determinants. It has become evident over the past few years that ESCs fluctuate among different levels of potency as a consequence of paracrine effects and cell-to-cell interactions that are not homogeneously regulated within current *in vitro* culture conditions [1,2,3]. The ESCs culture heterogeneity may be revealed by the expression of genes that distinctly mark ESCs subpopulations that are commonly defined metastates. Recent evidence reported that the ESCs metastate marked by Zscan4 (zinc finger and SCAN domain containing 4) is characterized by the remarkable potential to produce both embryonic and extra-embryonic cell lineages, therefore it is referred to as totipotent ESCs metastate [4,5,6,7]. The Zscan4 metastate is activated in about 3–5% of the ESC population at any given time and it is required to prevent senescence thus improving the quality of long-term culture. The Zscan4 metastate is enriched in high pluripotency culture conditions by the coordinated actions of extrinsic regulators, signaling pathways, and transcription factors. In particular, the cytokine Leukemia Inhibitory Factor (Lif) is implicated in the establishment of the Zscan4 ESCs metastate, while Phosphoinositide-3-kinase (PI3K) signaling has been reported to regulate Zscan4 expression [8]. Although Zscan4 ESC fluctuation is stabilized by high-pluripotency culture conditions obtained through Extracellular signal Regulated-Kinase (ERK) and Glycogen synthase kinase-3 (Gsk-3) signaling inhibition (2i) [9], the Zscan4 metastate is heterogeneously marked by *Gm12794* that defines a not-canonical pluripotency signature required for ESCs maintenance [10,11].

In the current study, we investigated how differentiation stimuli such as Dimethylsulfoxide (DMSO), Lif removal, and Retinoic Acid (*RA*) could affect Zscan4 metastate balance. Interestingly, we found that the Zscan4 metastate was negatively affected by Lif removal and by DMSO treatment, meanwhile it was significantly increased in RA culture condition. We have extended these results by evaluating RA induced Zscan4 metastate through global expression profile, and both self-renewal, and pluripotency capabilities. Our data showed that although the Zscan4 metastate retains self-renewal and pluripotency capabilities, it is also characterized by key markers of ectoderm lineage such as *cadherin20* (*Cdh20*), and *brain derived neurotrophic factor* (*Bdnf*). Consistently, the conditional ablation of the Zscan4 subpopulation is dispensable for both endoderm and mesoderm but is required for ectoderm lineage. In our opinion, the Zscan4 metastate enhanced by RA is primed to early ectoderm differentiation and represents a suitable opportunity to characterize key molecular signaling underlying the fluctuation between pluripotency maintenance, and early specification.

## Materials and Methods

### Cell culture

E14Tg2a.4 ES cells, derived from strain 129P2/OlaHsd were purchased from ATCC company and were cultured for two passages on gelatin-coated feeder-free plates and subsequently maintained in gelatin-coated six-well plates in complete ES medium: GMEM (Glasgow Minimum Essential Medium, Gibco), 15% FBS (EuroClone), 1,000 U ml-1 leukaemia inhibitory factor (LIF) (EuroClone), 1.0 mM sodium pyruvate (Invitrogen), 0.1 mM non-essential amino acids (Invitrogen), 2.0 mM L-glutamine (Invitrogen), 0.1 mM β-mercaptoethanol and 500 U ml-1 penicillin/streptomycin (Invitrogen). ESCs were incubated at 37°C in 6% CO2; medium was changed daily and cells were split every 2 to 3 days routinely. For differentiation, ESCs were plated in the medium supplemented with a combination of the following compounds: 1.5 μM all-trans RA, 5.0 μM BMS493, 1.0 μM BMS753 and 1.0 μM UVI2060. All experiments were performed at least three times.

### pGm12794-Strawberry vector construction

To generate the pGm12794-Strawberry vector, the Strawberry gene was amplified with the couple of primers *KpnI*-*AscI*-*EcoRV*-StrawF1 (5’-atggtgagcaagggcgaggagaataac-3’) and *BglII*-StrawR1 (5’-ctacttgtacagctcgtccatgccg-3’); the bgHpA poly(A) signal was amplified from the plasmid pL452 (from National Cancer Institute—Frederick) using the couple of primers *BglII*-pAf (5’-cttcttgacgagttcttctgagggg-3’) and *EcoRI*-*SalI*-pAr (5’-gttatattaagggttccgcaagc-3’). Both PCR products were cloned in pL452 using the *KpnI*-*BglII*-*EcoRI* restriction sites. Finally, a 5.0 kb region, upstream the ATG of the *Gm12794*, was amplified from the BAC bMQ299i11by PCR using the primers pRNIf (5’-ttcaaaggctgctagtggaagactg-3’) and pRNIr (5’-ataatttcaggctaagttttggaaattcc-3’) and was inserted in pCR-XL-TOPO (Invitrogen), from which was cut *MluI*-*EcoRV* and ligated in pL452-Strawberry digested *AscI*-*EcoRV*.

### Generation of pZscan4-Emerald/pGm12794-Strawberry cell line

The pZscan4-Emerald cells [5], a gift from Dr. Minoru S.H.Ko, were cultured for 2 passages on gelatin-coated feeder-free plates and subsequently maintained in gelatin-coated 6-well plates in complete ES medium: DMEM (Gibco); 15% FBS (EuroClone); 1000 U/ml leukemia inhibitory factor (LIF) (ESGRO, Chemicon); 1 mM sodium pyruvate; 0.1 mM non-essential amino acids (NEAA), 2 mM GlutaMAX, 0.1 mM beta-mercaptoethanol, and 500 U ml-1 penicillin/streptomycin. The number of 5×10^6^ cells in suspension were electroporated with 3 μg of linearized pGm12794-Strawberry and plated in gelatin-coated 100 mm dishes. Cells were selected with 250 μg/ml G418. ES cell colonies were picked on the 8th day, expanded, and frozen for further analysis.

### Flow cytometry and sorting pZscan4-Emerald and pGm12794-Strawberry ESCs

pZscan4-Emerald/pGm12794-Strawberry cells were fed at least 2 hours before harvesting by Trypsin (Gibco) and resuspended in complete ES medium containing 25 mM HEPES buffer. The cells were then FACS-sorted according to the fluorescent intensity of EMERALD or STRAWBERRY into complete ES medium containing HEPES. For qPCR analysis of FACS-sorted cells, total RNAs were collected immediately after sorting by TRIzol (Invitrogen) according to the manufactures instruction.

One microgram of total RNA was reverse-transcribed by Quantitec reverse transcription kit (Qiagen) according to the manufacturer’s instructions. qPCR analyses were performed using 7.5 ng cDNA per well in duplicate with the SYBR green master mix (Applied Biosystems) according to the manufacturer’s instructions. Reactions were run on QuantStudio 7 Flex system (Applied Biosystems). Fold induction was calculated and normalized by the ΔΔCt method. The gene-specific primers are available in S1 Table.

### RNA extraction, labeling and hybridization on the NIA 22K 60-mer oligo microarray

ESCs were sorted based on the presence and absence of Emerald fluorescence at culturing day 5, and mRNA was extracted from each subset using a Quickprep micro poly-A RNA Extraction Kit (Amersham Biosciences, Piscataway, NJ, USA) with linear acrylamide as a carrier (Ambion, Austin, TX, USA). The mRNA aliquots from each subset were labeled with Cy3 and Cy5 dyes by two-round linear amplification labeling reactions to make each cRNA target using a Fluorescent Linear Amplification Kit (Agilent Technologies, Palo Alto, CA, USA). A complete list of annotated gene content of the microarray can be found at the NIA mouse cDNA project web site (http://lgsun.grc.nia.nih.gov/cDNA/cDNA.html). Data are being submitted on Gene Expression Omnibus.

### Microarray data analysis

Intensity values of 21939 gene features per array were extracted from scanned microarray images using Feature Extraction 5.1.1 software (Agilent Technologies), which performs background subtractions and dye normalization. Text output was processed using an application developed in-house to perform ANOVA analysis (http://lgsun.grc.nia.nih.gov/ANOVA/). Intensities measured with a>50% error were replaced with missing values except for features with very low intensity. Surrogate values equal to mean error were inserted for values that were negative or less than the probe error. Pair-wise mean comparison was done using t-statistics and the false discovery rate (FDR = 15%) method. The small number of biological replications typical in expression profiling experiments results in a highly variable error variance, and this problem is usually addressed by log-ratio thresholds that require subjective decisions about biological significance, or by Bayesian adjustment of error variance that may still underestimate error variance and result in false positive results. To reduce false-positives, we opted for a very conservative error model in which the error variance used for estimating F-statistics for a feature is the maximum of the actual error variance and the average error variance in 500 features with similar average intensity. Statistical significance was determined using the false discovery rate FDR (= 15%) method. A scatter plot was created using the NIA microarray analysis tool (http://lgsun.grc.nia.nih.gov/ANOVA/).

### Cell signaling inhibition and Western Blot analysis

Specific inhibitors were used to probe the inhibition and attenuation effects on phosphorylation. ES cells were pre-treated for 90 minutes with 20 μM MEK1 inhibitor PD0325901. RA was added after 90 minutes of inhibitors for 24h. ES cells were pre-treated for 12h with 5 μM LY294002 and maintained in culture for 24h in presence of RA and LY294002. Because inhibitors were dissolved in dimethylsulfoxide (DMSO), control cells were similarly treated with DMSO.

Total protein was extracted with cell extraction buffer using the following formulation: 100 mM Tris pH7.4, 2 mM Na3VO4, 100 mM NaCl, 1% NP40, 1 mM EDTA, 1 mM NaF, 0.5% deoxycholate, 20 mM Na4P2O7, 1 mM PMSF and 1X Protease Inhibitor Cocktail (Sigma). Protein concentrations were determined using Bio-Rad protein assay kit according to the manufacturer’s instructions. Twenty microgram protein was separated on SDS-PAGE and transferred onto nitrocellulose membrane. The following primary antibodies were used: rabbit anti-phospho-ERK1/ERK2 (1:1000; Cell Signaling), rabbit anti-total ERK1/ERK2 (1:1000; Cell Signaling), rabbit anti-Vinculin (1:1000; Cell Signaling), rabbit anti-Nanog (1:1000; Abcam) and mouse anti-Actin (1:1000; Sigma). The membranes were incubated with antibodies to specific proteins followed by incubation with HRP-conjugated anti-rabbit IgG or anti-mouse IgG (1:2500; Santa Cruz Biotechnology).

### Cell ablation strategy

To generate the pGm12794-TK vector, a DNA fragment containing the thymidine kinase (TK) coding sequence completed of a poly(A) signal was amplified with the couple of primers *HpaI*-TKf (5’-agcgcgtatggcttcgtacc-3’) and *SalI*-TKr (5’-cttgataccccacgcaacgc-3’). This fragment was inserted in the pGm12794-Strawberry digested *EcoRV*-*SalI*, replacing the Strawberry-pA sequence. All the passages of the plasmids construction were verified by sequence analysis.

## Results

### Zscan4 is induced in ESC cultured with RA

**Fig 1:**
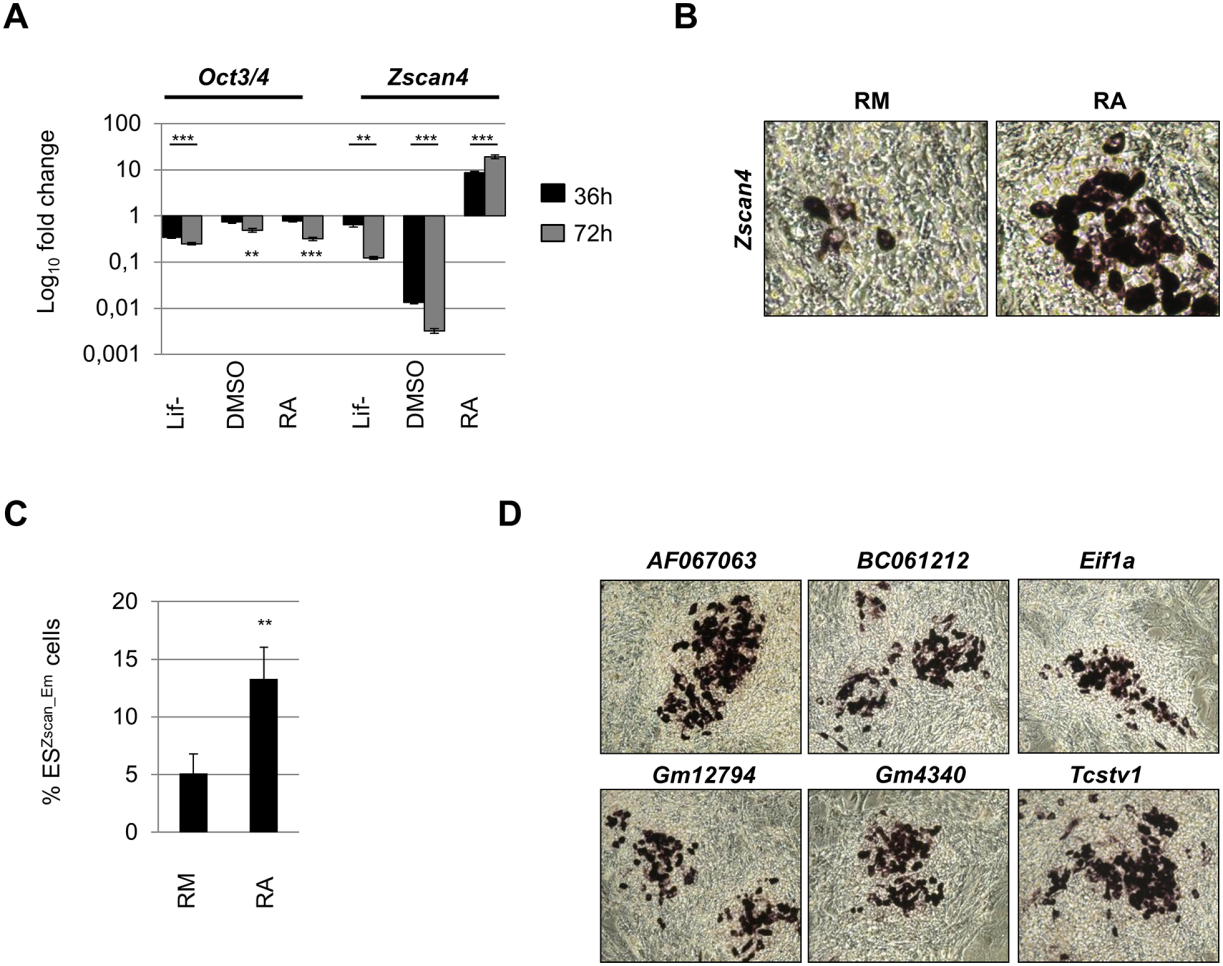
RA induces Zscan4 ESC metastate. (A) ESCs were cultured for 36 and 72 h in differentiating conditions: Lif-, 1% DMSO and 1.5 μM RA. The mRNA expression levels were assessed by qRT-PCR and normalized to RM condition. The average and SD of duplicate samples from each of three independent biological replicates are shown: **, p < .01; ***, p < .001, in a Student’s *t* test. (B) *Zscan4* expression pattern in ESCs cultures by RNA in situ hybridization upon 5 days of treatment in RM or RA. RNA ISH showed “spotted” patterns on ESC colonies (40x). (C) Percentage of RM-Zscan4^+^ and RA-Zscan4^+^ cells was evaluated by flow cytometry analyses. The mean % ES^Zscan4_Em^ cells ± SD of three independent experiments is presented with statistical analysis performed using Student’s *t* test (**, p < .01). (D) Distribution of *Zscan4* related gene signature in ESC cultures by RNA in Situ hybridization showed “spotted” patterns on ESC colonies. Most representative colonies were magnified (20x) to show the detailed staining patterns.

We investigated how the Zscan4 metastate is affected during ESCs differentiation. For this purpose, we cultured ESCs in three differentiating culturing media: first, we used regular medium (hereafter *RM*) without *Leukemia inhibitory factor* (hereafter *Lif-*), a cytokine required for ESC self-renewal; the second culture medium was obtained adding 1% of *Dimethylsulfoxide* (hereafter *DMSO*), which is a chemical inducer of ESCs differentiation across all three germ layers [12]; the third culture condition was RM supplemented with *Retinoic Acid* at 1.5 μM (hereafter *RA*), which is a crucial signaling molecule during embryonic morphogenesis and ESC differentiations [13,14]. ESCs differentiation induction was evaluated based on the ability of culturing media to reduce expression of *Oct3/4* that it is canonical marker of pluripotency. *Oct3/4* expression was compared among the three differentiating conditions both at 36 hours (h), and at 72h through quantitative PCR (qRT-PCR) analyses. As expected *Oct3/4* expression was downregulated in all culturing media (Fig 1A). The Zscan4 ESCs metastate was evaluated based on the expression of *Zscan4*. Compared to RM, *Zscan4* expression was reduced in *Lif-* (about 2 folds at 36h, and about 8 folds at 72h), and in *DMSO* (about 74 folds at 36h and about 300 folds at 72h). The expression of Zscan4 remarkably it was upregulated in *RA* (about 9 folds at 36h, and about 20 folds at 72 h) (Fig 1A). We evaluated whether *Zscan4* induction reflects an increase of Zscan4 metastate percentage within ESC cultures through RNA *in situ* hybridization (ISH) (Fig 1B). RNA *ISH* results showed “spotted” patterns of Zscan4 expression on ESCs colonies that are consistent with the increase of the Zscan4 metastate. To quantitatively evaluate the Zscan4 metastate increase, we employed transgenic ESCs line (ES^*Zscan4_Em*^ cells) in which the expression of *Zscan4* is marked by green fluorescence of Emerald reporter (S1A Fig) enabling us to characterize RA-Zscan4^+^ in living cell cultures [5]. In particular, we cultured ES^*Zscan4_Em*^ cells both in RM and in RA media, and compared the percentage of fluorescent cells (Zscan4^+^) through cytofluorimetry analyses. The percentage of green cells measured in ESCs culture at 72h was about 5% in RM (hereafter RM-Zscan4^+^), and it was about 14% in RA (hereafter RA-Zscan4^+^) (Fig 1C and S1 Video). Furthermore, we performed RNA ISH also on *AF067063*, *BC061212*, *Eif1a*, *Gm12794*, *Gm4340*, and *Tcstv1* because altogether those genes define the Zscan4 metastate molecular signature [10]. The results revealed mosaic in colony expression patterns (Fig 1D) that were consistent with the Zscan4 metastate enrichment, and strengthen our conclusion that RA culture condition enhances ESC Zscan4 metastate fluctuation [6,7].

### RA induction of Zscan4 metastate is RAR dependent

**Fig 2:**
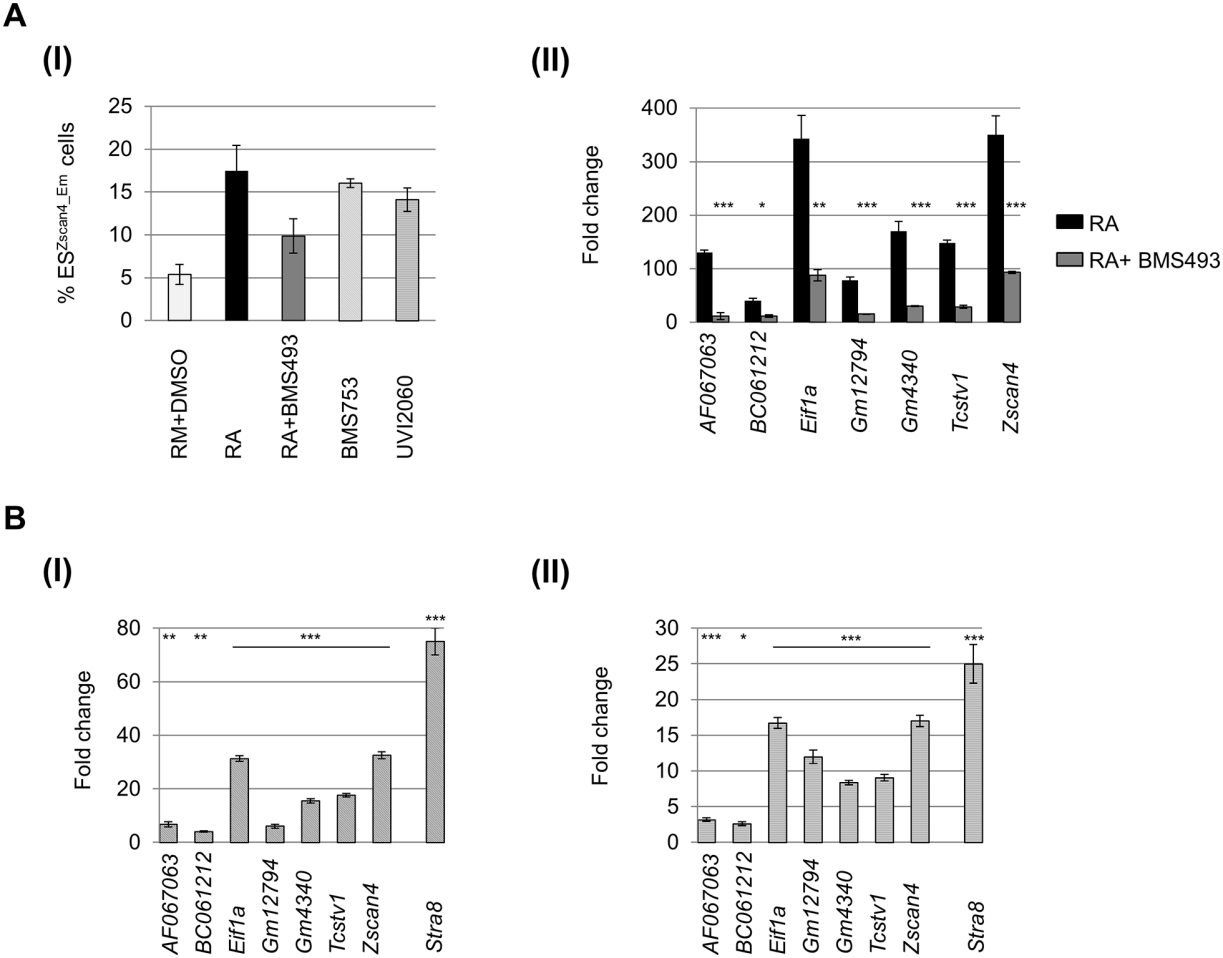
Zscan4 metastate induction requires canonical RAR signaling. (A) (I) The percentage of ES^Zscan4_Em^ cells was assessed on ESCs cultured for 3 days in: RM+DMSO; RM supplemented with RA; RA plus BMS493; BMS753, and UVI2060. The mean % ES^Zscan4_Em^ cells ± SD of two independent experiments is shown. (II) The mRNA expression levels were assessed on ESCs treated with RA and RA plus BMS493 by qRT-PCR and normalized to RM+DMSO condition. The average and SD of duplicate samples from each of three independent biological replicates are shown: *, p < .05; **, p < .01; ***, p < .001, in a Student’s *t* test. B (I) The mRNA expression levels were assessed on ESCs treated with BMS753 by qRT-PCR and normalized to RM+DMSO condition. The average and SD of duplicate samples from each of three independent biological replicates are shown: **, p < .01; ***, p < .001, in a Student’s *t* test. (II) The mRNA expression levels were assessed on ESCs treated with UVI2060 by qRT-PCR and normalized to RM+DMSO condition. The average and SD of duplicate samples from each of three independent biological replicates are shown: *, p < .05; ***, p < .001, in a Student’s *t* test.

In order to characterize RA signaling involved in *Zscan4* induction, we assayed the requirement of RA receptors (RARs). First, we antagonized the three RAR isotypes (RARα, RARβ and RARγ) by the addition of BMS493 in RA culturing medium, a compound known to antagonize the three RAR isotypes [15]. We evaluated the effects of BMS493 on the RA-Zscan4^+^ metastate by comparing the percentage of Zscan4^+^ cells in Emerald transgenic cell line cultured in RA with and without RAR antagonists. The ES^*Zscan4_Em*^ cells fluorescence was measured by flow cytofluorimetry analyses on day3, and, showed that the RA-Zscan4^+^ metastate percentage was about 18% in RA and it was reduced to about 10% in RA with BMS493 (Fig 2AI). These data were further confirmed by qRT-PCR analysis conducted on the genes that mark Zscan4 metastate (Fig 2AII). In particular, RA inductions of *AF067063*, *BC061212*, *Eif1a*, *Gm12794*, *Gm4340*, and *Tcstv1* were strongly reduced by BMS493 treatment. Second, we further dissected the RA signaling requirement using RAR agonists [16]. BMS753 is a selective agonist of RARα, and UVI2060 is an agonist of both RARβ, and RARγ. In particular, we compared the Zscan4^+^ cell percentage among ESCs cultured in: RM, RM+BMS753, and RM+UVI2060 by cytofluorimetry analyses. We found that Zscan4^+^ percentage in RM (about 6%) was increased up to 15% in RM+BMS753, and up to 14% in RM+UVI2060 (Fig 2AI). In order to validate those data, we also assayed the expression of the Zscan4 metastate signature (*AF067063*, *BC061212*, *Eif1a*, *Gm12794*, *Gm4340*, and *Tcstv1*). Consistently with cytofluorimetry results, we found that both BMS753 and UVI2060 treatments induced the Zscan4 metastate gene signature (Fig 2BI and 2BII). The assay efficiency was measured based on *Stra8* expression, since it was previously reported to be specifically induced by RA [17]. In conclusion, our data showed that RA-Zscan4^+^ induction was mediated through RARs signaling.

### RA-Zscan4 activation is PI3K dependent

**Fig 3:**
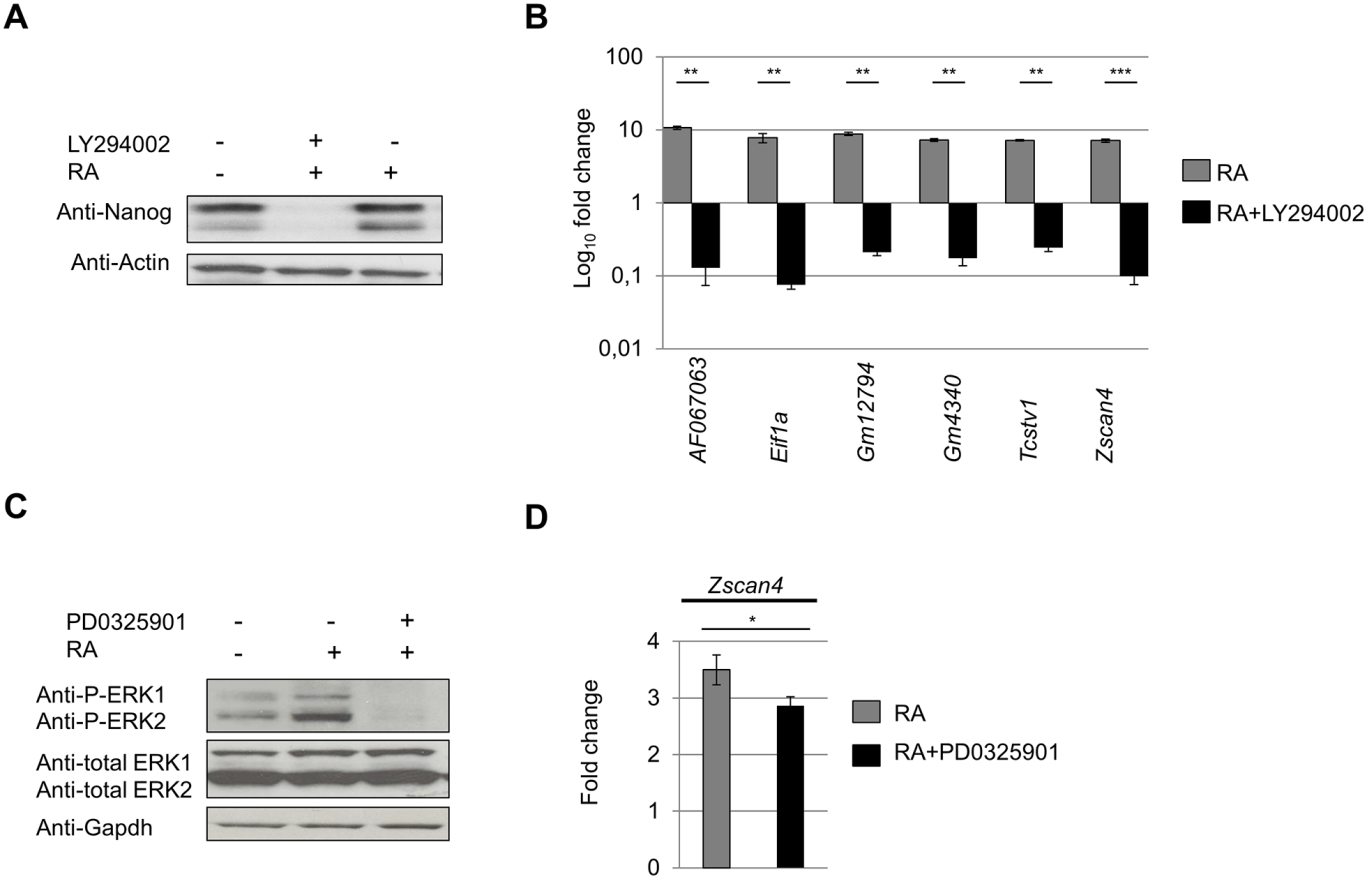
PI3 Kinase activation regulates Zscan4 metastate induction. (A) Western Blot analysis on ESCs treated with either RA or RA with 5.0 μM LY294002 for 12h. NANOG was considered the positive control for treatment evaluation. (B) The mRNA expression levels were assessed by qRT-PCR and normalized to RM condition. The average and SD of duplicate samples from each of three independent biological replicates are shown: **, p < .01; ***, p < .001, in a Student’s *t* test. (C) Western Blot analysis on ESCs were treated with either RA or RA with 20 μM PD0325901 for 12h. ESCs treatment with RA increased pERK1/ERK2, whereas PD treatment hampered pERK1/ERK2. (D) The mRNA expression levels are assessed by qRT-PCR and normalized to RM condition. The average and SD of duplicate samples from each of three independent biological replicates are shown: *, p < .05, in a Student’s *t* test.

Recent reports show that phosphoinositide-3-kinase (PI3K) signaling, known to be crucial for self-renewal maintenance, is required to establish the Zscan4^+^ pluripotent metastate in RM [8,18]. Here, we investigate whether PI3Ks is also involved in RA-dependent Zscan4^+^ induction. ESCs were cultured for 12 hours with LY294002, an inhibitor of PI3K. We validated the effectiveness of PI3K inhibition treatment by addressing NANOG expression, which is a known self-renewal marker positively regulated by PI3K [8]. The immunoblot analysis using NANOG antibody confirmed that continuous exposure to LY294002 suppressed the expression of the pluripotency markers NANOG (Fig 3A). In the same culture conditions, we evaluated the effects of PI3K inhibition on the expressions of the Zscan4-related molecular signature through qRT-PCR. Expressions of *AF067063*, *Eif1a*, *Gm12794*, *Gm4340*, *Tcstv1*, and *Zscan4* were dramatically dropped in ESCs cultures with RA with LY294002 (Fig 3B). We also examined involvement of ERK signaling that, in contrast to PI3Ks, promotes ESCs differentiation. As the Mek/Erk pathway is also a regulatory of early differentiation and was first considered for self-renewal due to its constitutive activity in ESC cultures, we assessed its impact on Zscan4 expression [19,20]. In particular, to evaluate the correlation between ERKs and RA-Zscan4^+^ induction, we cultured ESCs in RA with and without PD0325901, a selective ERKs signaling inhibitor. The Western Blot analysis showed that RA activated ERKs by increasing phosphorylation of ERK1, and ERK2 (Fig 3C). Although ERKs activation was efficiently suppressed by PD0325901 treatment, *Zscan4* expression did not change (Fig 3D). Finally, we demonstrated that PI3Ks and not ERKs signaling activation was required in RA-induced Zscan4^+^ activation.

### RA-Zscan4^+^ retains high pluripotency molecular signature and self-renewal capabilities

**Fig 4:**
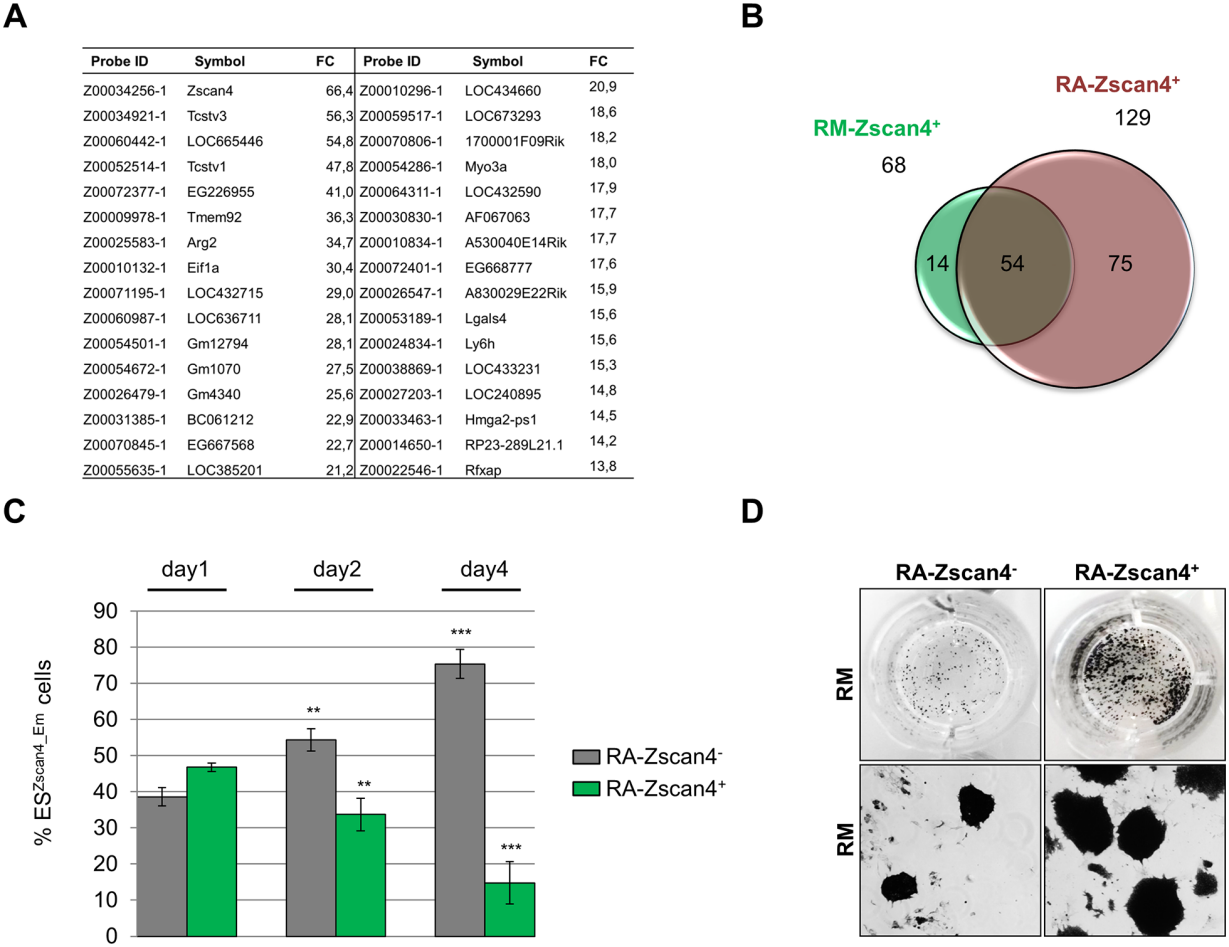
RA-Zscan4^+^ cells molecular and cellular characterization. (A) The global gene expression profiles between RA-Zscan4^+^ and RA-Zscan4^-^ cell populations by DNA chip microarray hybridization. List of RA-Zscan4^+^ probes upregulated more than 20-fold compared to RA-Zscan4^-^. (B) RA-Zscan4^+^ cells expression versus RM-Zscan4^+^. (C) RA-Zscan4^+^ and RA-Zscan4^-^ cells were collected, separated by FACS and plated in RM. The mean % RA-Zscan4^+^ or RA-Zscan4^-^ cells ± SD of three independent experiments is presented with statistical analysis performed using Student’s *t* test (**, p < .01; ***, p < .001). (D) RA-Zscan4^+^ and RA-Zscan4^-^ cells were collected and separated by FACS, plated in RM and were characterized based on AP-positive colonies after 5 days in RM (*n* = 3).

Since retinoic acid is a known cellular morphogen it was an intriguing question to examine whether RA-Zscan4^+^ subpopulation represents a differentiation state. In particular, we cultured ES^*Zscan4_Em*^ cells in RA for 5 days, collected homogeneous RA-Zscan4^+^ and RA-Zscan4^-^ populations using fluorescence activated cell sorting (FACS), and then we compared their global gene expression profiles through DNA microarray hybridization methodology. Bioinformatic analyses revealed that RA-Zscan4^+^ expressed 129 genes 3-folds more than RA-Zscan4^-^ (Fig 4A, S2 Table). To assess how similar the Zscan4 metastate induced in RA was to the Zscan4 metastate in RM, we compared 129 transcripts enriched in RA-Zscan4^+^ versus 68 genes specifically enriched in RM-Zscan4^+^. Remarkably, the RA-Zscan4^+^ signature contained 54 out of 68 genes enriched in RM-Zscan4^+^ corresponding to about 80% of molecular signature (Fig 4B). These analyses suggested that RA induced subpopulation marked by Zscan4 expression was closely related to known high pluripotent ESCs metastate based on their transcription profile. The further analysis of known genes enriched only in RA-Zscan4^+^ and not in RM-Zscan4^+^ revealed that only 34 out of 75 have known gene symbols and include among others key factors that are known to be specifically required for neuronal lineage specification (*Bdnf*, and *Cdh20*) [21,22]. On the other hand among genes that are specific of RM-Zscan4^+^ and not revealed in RA-Zscan4^+^, 9 out of 14 have known gene symbols and among these there was *Tbx3* that was recently described in published work as playing a role in promoting transition of ESC toward Zscan4^+^ metastate [23,24]. Altogether these data prompted us to evaluate whether RA-Zscan4^+^ could be primed to differentiation. In particular, we homogeneously collected RA-Zscan4^+^ and RA-Zscan4^-^ on day 5 through FACS, then we plated in RM, and characterized both the expressions of pluripotency markers, and the morphology. First, the qRT-PCR expression analyses revealed that RA-Zscan4^+^ cultured in RM were enriched in pluripotency markers compared RA-Zscan4^-^ (S1B Fig). Second, RA-Zscan4^+^ plated in RM gradually restored RA-Zscan4^-^ cell population, going from 100% on day 0 to about 12% on day 4 (Fig 4C), giving rise to ESC colonies that were expressing high level of *alkaline phosphatase* (AP), a known marker of colony pluripotency (Fig 4D). Altogether, these data suggested that Zscan4^+^, although induced by RA, maintained pluripotency potential.

### RA induced Zscan4 metastate transition toward ectoderm derivatives

**Fig 5:**
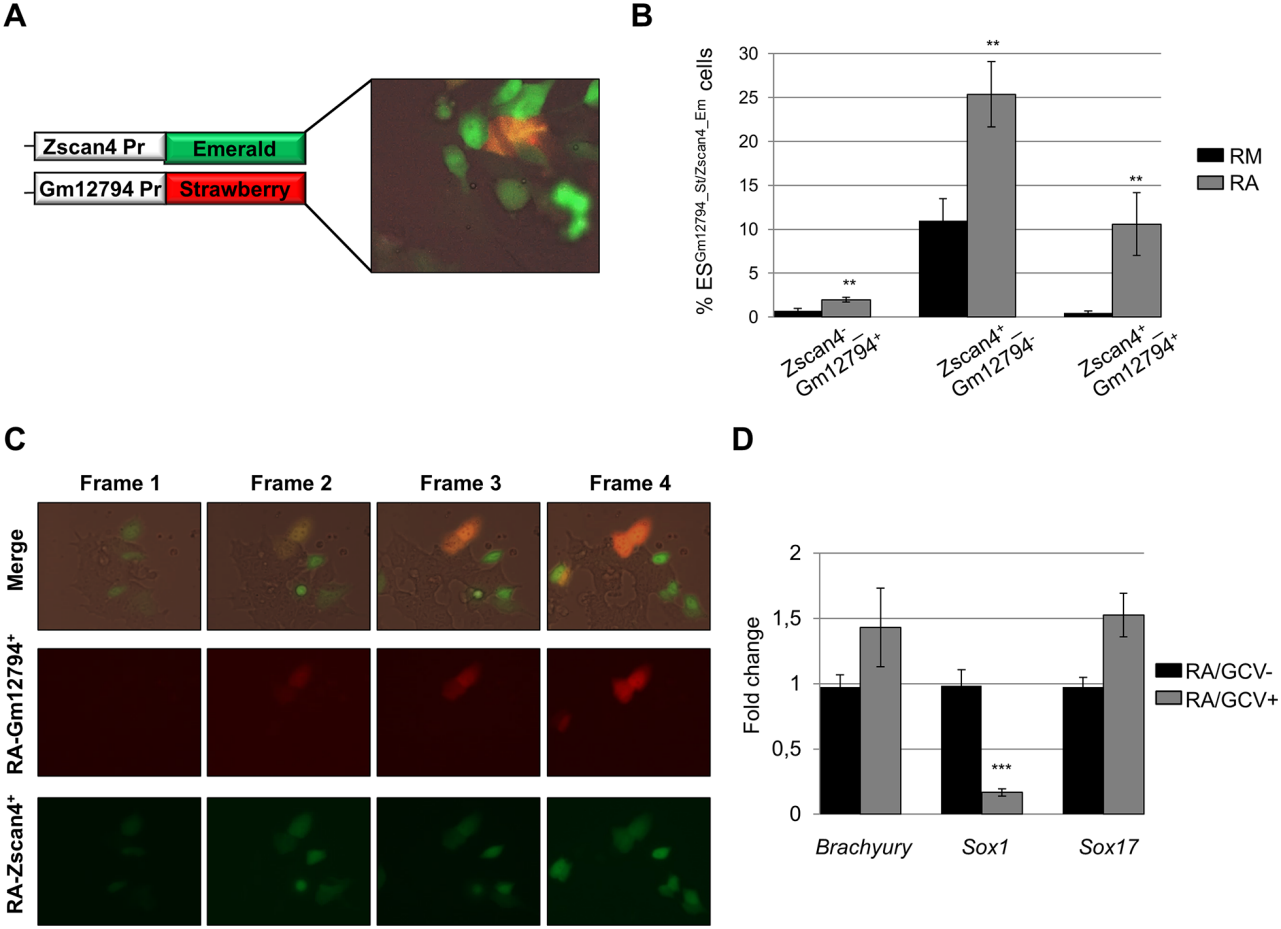
RA induces Zscan4 metastate transition. (A) *Zscan4* and *Gm12794* expressions were visualized by Emerald and Strawberry reporter respectively. (B) Percentage analyses of ES^Gm12794_St/Zscan4_Em^ cells upon 3 days of treatment in RM or RA by flow cytometry. The mean % ES^Gm12794_St/Zscan4_Em^ cells ± SD of three independent experiments is presented with statistical analysis performed using Student’s *t* test: **, p < .01. (C) Cell live imaging reported in 4 time frames of 2 hour each. (D) ES^Gm12794_HSVTK^ cell line and control E14Tg2a.4 cultured in media supplemented with RA in presence or absence of GCV (2.0 μM, Sigma). The mRNA expression levels were assessed by qRT-PCR and normalized to RA/GCV- condition. The average and SD of duplicate samples from four independent biological replicates are shown: ***, p < .001, in a Student’s *t* test.

The RA-Zscan4^+^ molecular signature included *Gm12794* is a novel member of the Prame family, known to be involved in RA signaling [25,26,27,28]. To characterize the effects of RA on *Gm12794*, we generated a transgenic ESC line in which *Zscan4* and *Gm12794* promoters regulated the expressions of Emerald and Strawberry fluorescent reporters respectively (hereafter named ESC^*Gm12794_St / Zscan4_Em*^) (Fig 5A). This ESC cell line enabled us to quantify the percentage of ESCs expressing either *Gm12794* and/or *Zscan4* in the same culture conditions. The percentage measures were done in ESCs cultured in RM on day3 and in RA on day3 through fluorescent cytofluorimetry. The results showed that compared to RM culture conditions, RA induced Zscan4^+^/Gm12794^-^ metastate less than 3 folds (≈10% in RM, and ≈25% in RA; n = 3), Zscan4^-^/ Gm12794^+^ metastate was induced about 3 folds (≈0.5% in RM and ≈1.5% in RA; n = 3), and Zscan4^+^/ Gm12794^+^ more than 30 folds (≈0.3% in RM and ≈10% in RA; n = 3) (Fig 5B). To elucidate whether the Zscan4^+^/Gm12794^+^ metastate increase depends on Zscan4^+^ metastate induction, we set up time-lapse live imaging visual inspection on ESC^*Gm12794_St / Zscan4_Em*^ cell line. We observed that the Gm12794^+^ metastate derived from the Zscan4^+^ subpopulation (Fig 5C, and S1 Video). Altogether these data revealed that RA enhance the Zscan4 metastate transition to Gm12794 metastate.

We investigated whether Gm12794 metastate transition is associated to RA induced ESCs differentiation. For this purpose we selectively ablated Gm12794^+^ cell using herpes simplex 1 virus thymidine kinase (HSV-TK) system. Briefly, HSV-TK expression renders cells sensitive to the nucleoside analog ganciclovir (GCV). We used an ESC transgenic line in which the expression of HSV-TK was under the control of *Gm12794* promoter. The ESC^*Gm12794_HSVTK*^ were cultured in RA culturing media with GCV (GCV^+^), and without GCV (GCV^-^) and on day 3, and further characterized for the expression of ectoderm (*Sox1*), endoderm (*Sox17*, *and Foxa2*), and mesoderm (*Brachyury*, *and Gata2*) markers. The quantitative marker’s expression analyses between GCV^+^ and to GCV^-^ revealed that *Brachyury*, *Foxa2*, *Gata2*, *and Sox17* were enriched, meanwhile *Sox1* was strongly downregulated (Fig 5D and S1C Fig). In conclusion, Gm12794^+^ ESC metastate ablation hampered early ectoderm ESCs differentiation induced by RA.

## Discussion

Embryonic Stem Cells (ESCs) cultured in standard conditions exhibit expression heterogeneity for several transcription factors functionally linked to both pluripotency, and differentiation state of individual cells. An emerging theme is that cellular heterogeneity represents ESC fluctuations among metastates that play an important role in self-renewal maintenance. In particular, ESCs metastate marked by *Zscan4* expression define high pluripotent ESC metastate, known also as 2C-like state because it is marked by a panel of genes that are specifically expressed during 2 cell preimplantation development stage [5]. The Zscan4 metastate represents a fluctuating subpopulation that in average is about 5% of the ESC population in regular culture conditions [5], and is characterized by the potential to produce both embryonic and extra-embryonic cell derivatives [6]. In this work, we evaluated the Zscan4 metastate upon ESC differentiating treatments obtained by modifying regular culture mediums by removal of Lif, or by the addition of two morphogens such as DMSO, and RA.

Our data showed that RA culture condition is very strongly inducing the enrichment of Zscan4 metastate up to about 20% of the cell population. The RA effect on Zscan4 metastate enrichment is not dosage dependent and it occurs at low concentration (S1D Fig). In our study, we used RA specific agonists, and antagonists to investigate the roles of retinoid receptors in the induction of Zscan4 metastate. Our results revealed that induction of Zscan4 metastate by retinoids is mediated by all three RA receptors (RARs). The most common mechanism of action of these receptors involves their binding to RA response elements (RARE) in the promoters of retinoid-responsive genes. However, we found that the promoters of the genes that characterize the global expression profile of RA-Zscan4 metastate did not present RARE (*data not shown*), and therefore may be induced either by RARE-independent mechanism or as response to early RARE-dependent genes.

**Fig 6:**
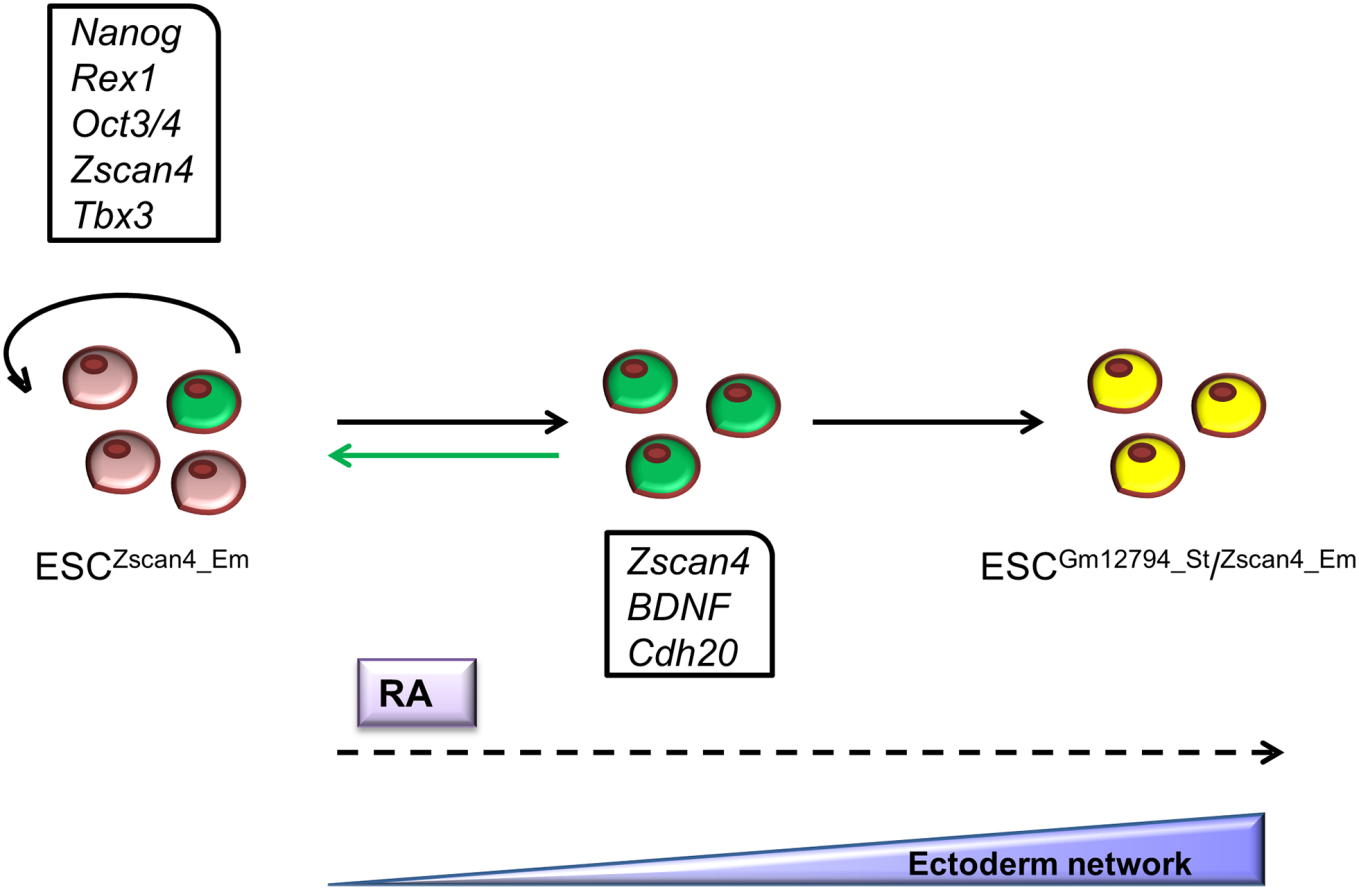
Model the activation of RA-Zscan4^+^ metastate during ESC differentiation through RA treatment.

The global gene expression comparison between RM-Zscan4 and RA-Zscan4 revealed significant transcriptome overlap (Fig 4B) that could be explained suggesting a common origin. Consistently with this hypothesis, the cell live imaging analysis showed that upon the addition of RA, RM-Zscan4 cells were induced to active division. Based on genes that are specifically enriched either in RM-Zscan4 or in RA-Zscan4, we summarized a cellular transition model in Fig 6. Briefly, *Tbx3*, known to be a direct inducer of the Zscan4 is more expressed in RM-Zscan4 metastate than in the RA-Zscan4; while *Gm12794* a member of the Prame family [27], *Bdnf* and *Cdh20* that are key markers of ectoderm lineage [23,24] are among the genes significantly enriched in RA-Zscan4. Altogether these data suggest that RA-Zscan4 metastate is primed to differentiate toward ectoderm lineage.

This hypothesis is also further supported by ablation cell lineage assessments. In particular, our data revealed that removing the RA-Zscan4 population during RA differentiation specifically hampers ectoderm, but not endoderm or mesoderm derivatives (S1C Fig).

Remarkably, RA-Zscan4 homogeneously isolated population once plated in regular medium revert to ESC that express canonical markers of pluripotency, and retained self-renewal capability by giving rise to colonies positive for the pluripotency marker AP. Altogether, we conclude that although the Zscan4 metastate enhanced by RA is primed to ectoderm lineage it is not fully differentiated. In our opinion, this research provides insights to extend the molecular characterization of self-renewal and differentiation balancing mechanisms, reducing ambiguous high pluripotency metastate definition. Further extensive analysis of this retinol signaling cascade could enhance our ability to understand, and regulate stem cell differentiation in the context of ectoderm embryogenesis.

## Supporting Information

S1 Fig (A) Zscan4 induction by RA treatment visualized by Emerald reporter. (B) RA-Zscan4^+^ and RA-Zscan4^-^ were collected through FACS and plated in RM. The mRNA expression levels were assessed by qRT-PCR and normalized to RA-Zscan4^-^ condition. The average and SD of duplicate samples from two independent biological replicates are shown. (C) ES^Gm12794_HSVTK^ cell line and control E14Tg2a.4 were cultured in media supplemented with RA in presence or absence of GCV (2.0 μM, Sigma). The mRNA expression levels were assessed by qRT-PCR and normalized to RA/GCV- condition. The average and SD of duplicate samples from four independent biological replicates are shown: *, p < .05, in a Student’s t test. (D) qRT-PCR on ES cell cultured in RA at different concentrations. The average and SD of samples from three independent biological experiments are shown: ***, p < .001, in a Student’s t test.
(TIF)

S1 Table Gene-specific primers used for qRT-PCR. (TIF)

S2 Table Pair-wise gene expression profiles. List of genes enriched in RA-Zscan4^+^ compared to RA-Zscan4^-^.
(PDF)

S1 Video Time-lapse live imaging. Transgenic ESCs line in which Zscan4 promoter drives green fluorescent reporter (frame time: 30 minutes).
(AVI)
