# Supplementary figures and images for: Retinoic Acid Specifically Enhances Embryonic Stem Cell Metastate Marked by Zscan4
Source: PLoS One. 2016 Feb 3;11(2):e0147683. doi: 10.1371/journal.pone.0147683 (PMC4740454; doi:10.1371/journal.pone.0147683)

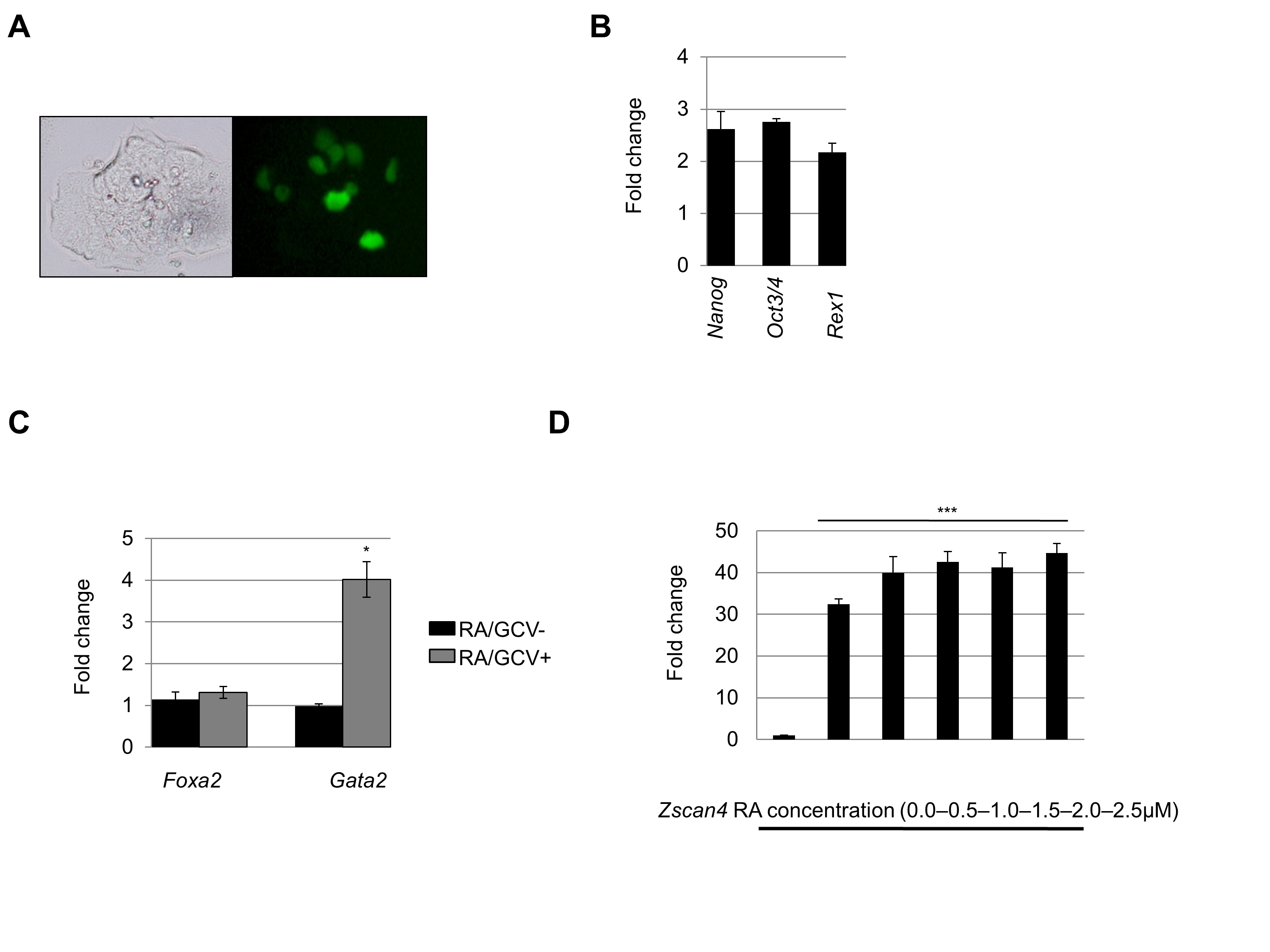

Supplement: S1 Fig — (A) Zscan4 induction by RA treatment visualized by Emerald reporter. (B) RA-Zscan4+ and RA-Zscan4- were collected through FACS and plated in RM. The mRNA expression levels were assessed by qRT-PCR and normalized to RA-Zscan4- condition. The average and SD of duplicate samples from two independent biological replicates are shown. (C) ESGm12794_HSVTK cell line and control E14Tg2a.4 were cultured in media supplemented with RA in presence or absence of GCV (2.0 μM, Sigma). The mRNA expression levels were assessed by qRT-PCR and normalized to RA/GCV- condition. The average and SD of duplicate samples from four independent biological replicates are shown: *, p < .05, in a Student’s t test. (D) qRT-PCR on ES cell cultured in RA at different concentrations. The average and SD of samples from three independent biological experiments are shown: ***, p < .001, in a Student’s t test. (TIF) [file pone.0147683.s001.tif]

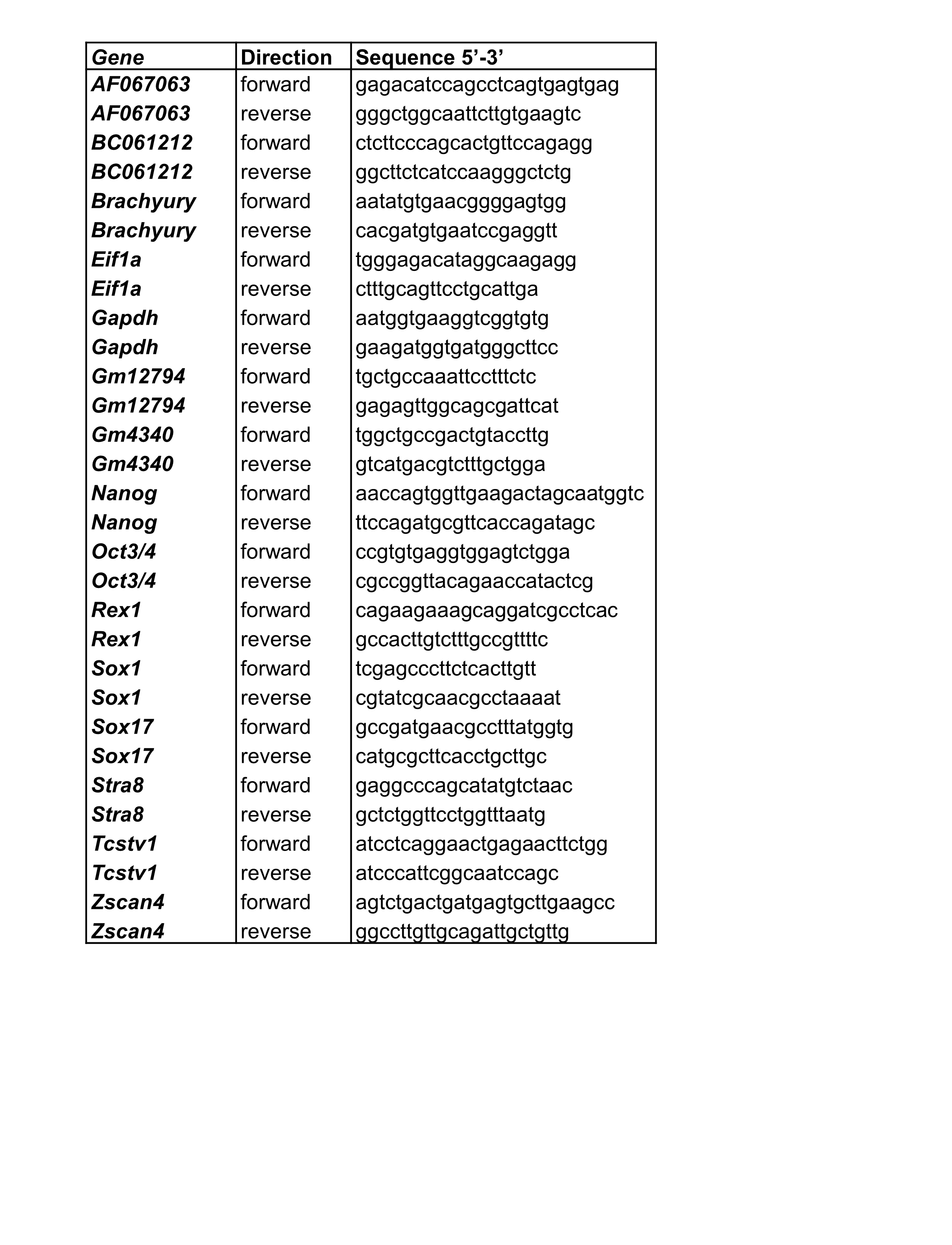

Supplement: S1 Table — (TIF) [file pone.0147683.s002.tif]
