# Supplementary material for: Retinoic Acid Specifically Enhances Embryonic Stem Cell Metastate Marked by Zscan4
Source: PLoS One. 2016 Feb 3;11(2):e0147683. doi: 10.1371/journal.pone.0147683 (PMC4740454; doi:10.1371/journal.pone.0147683)
Supplement: S2 Table — List of genes enriched in RA-Zscan4+ compared to RA-Zscan4-. (PDF) [file pone.0147683.s003.pdf]

Probe ID	Mean(Zscan4_RA_Em+)	Mean(Zscan4_RA_Em-)	Symbol	FC
Z00034256-1	5,5253	3,70341	Zscan4	66,4
Z00034921-1	5,0004	3,2496	Tcstv3	56,3
Z00060442-1	5,2807	3,5417	LOC665446	54,8
Z00052514-1	4,6523	2,9733	Tcstv1	47,8
Z00072377-1	4,8702	3,25761	EG226955	41,0
Z00009978-1	4,3346	2,7742	Tmem92	36,3
Z00025583-1	3,8719	2,3317	Arg2	34,7
Z00010132-1	4,959	3,47581	Eif1a	30,4
Z00071195-1	4,6089	3,1462	LOC432715	29,0
Z00060987-1	4,9258	3,47641	LOC636711	28,1
Z00054501-1	3,7799	2,331	Gm12794	28,1
Z00054672-1	4,1987	2,75981	Gm1070	27,5
Z00026479-1	5,4245	4,0164	Gm4340	25,6
Z00031385-1	4,3201	2,9605	BC061212	22,9
Z00070845-1	5,1497	3,7936	EG667568	22,7
Z00055635-1	4,3574	3,031	LOC385201	21,2
Z00010296-1	4,0298	2,7097	LOC434660	20,9
Z00059517-1	4,0152	2,7454	LOC673293	18,6
Z00070806-1	4,4783	3,2188	1700001F09Rik	18,2
Z00054286-1	3,9061	2,64991	Myo3a	18,0
Z00064311-1	3,6907	2,43741	LOC432590	17,9
Z00030830-1	4,1826	2,9338	AF067063	17,7
Z00010834-1	3,9168	2,66811	A530040E14Rik	17,7
Z00072401-1	3,2273	1,9828	EG668777	17,6
Z00026547-1	3,2094	2,00891	A830029E22Rik	15,9
Z00053189-1	3,7657	2,57231	Lgals4	15,6
Z00024834-1	4,3971	3,2039	Ly6h	15,6
Z00038869-1	3,44	2,25641	LOC433231	15,3
Z00027203-1	3,4953	2,32391	LOC240895	14,8
Z00033463-1	4,6287	3,4666	Hmga2-ps1	14,5
Z00014650-1	3,9762	2,82431	RP23-289L21.1	14,2
Z00022546-1	4,7973	3,6567	Rfxap	13,8
Z00025315-1	5,0109	3,8785	Pif1	13,6
Z00000661-1	3,3069	2,1912	Mgll	13,1
Z00050009-1	4,0282	2,91491	1700066J24Rik	13,0
Z00056063-1	4,1211	3,0164	EG666169	12,7
Z00032628-1	3,7256	2,62111	Cdh20	12,7
Z00057246-1	4,0682	2,977	LOC381535	12,3
Z00060458-1	3,9922	2,90641	Gm428	12,2
Z00026897-1	4,2001	3,1296	2310065F04Rik	11,8
Z00070299-1	5,1438	4,08731	3930401B19Rik	11,4
Z00058452-1	3,8351	2,79401	LOC669397	11,0
Z00030644-1	3,4158	2,38371	C130092O11Rik	10,8
Z00056563-1	3,7156	2,7147	Cand1	10,0
Z00054259-1	3,4261	2,43691	Lmx1a	9,8
Z00012520-1	4,8328	3,87821	Whsc2	9,0
Z00023692-1	3,8189	2,8669	Cpb2	9,0
Z00059946-1	3,9393	2,9931	Gm854	8,8
Z00070929-1	2,7187	1,7746	Gm257	8,8

Z00037504-1	3,8365	2,8953	Hspa1a	8,7
Z00058951-1	4,0608	3,1257	BC080695	8,6
Z00052446-1	3,6852	2,75641	Lonrf3	8,5
Z00031258-1	3,3013	2,3737	EG666099	8,5
Z00054412-1	3,3925	2,4959	1810062G17Rik	7,9
Z00031058-1	3,5221	2,63011	D5Ertd577e	7,8
Z00067545-1	4,2148	3,3378	LOC627530	7,5
Z00032758-1	3,296	2,44451	LOC226030	7,1
Z00072723-1	3,3306	2,481	LOC631120	7,1
Z00073053-1	4,5929	3,7467	Drr2	7,0
Z00059740-1	3,0357	2,18961	LOC673230	7,0
Z00072895-1	3,1366	2,2952	LOC625360	6,9
Z00030445-1	4,0076	3,16951	OTTMUSG0010537	6,9
Z00072933-1	3,2963	2,46561	LOC673656	6,8
Z00047227-1	3,4804	2,65	Zfp809	6,8
Z00020544-1	3,3661	2,578	Cacna1s	6,1
Z00023243-1	4,1061	3,32741	Ercc4	6,0
Z00034203-1	3,3613	2,58951	4631416L12Rik	5,9
Z00060019-1	3,9836	3,215	LOC673289	5,9
Z00060430-1	3,1281	2,37121	Ankrd22	5,7
Z00066847-1	4,1458	3,401	LOC619649	5,6
Z00056514-1	3,0214	2,28181	LOC215996	5,5
Z00026871-1	3,2971	2,55791	B020004J07Rik	5,5
Z00010220-1	2,4878	1,7497	B020006M18Rik	5,5
Z00058056-1	4,1593	3,42241	EG624262	5,5
Z00053533-1	3,9962	3,2657	Hist1h4i	5,4
Z00009042-1	4,036	3,30671	Fbxo15	5,4
Z00030855-1	3,1697	2,4464	LOC545920	5,3
Z00027180-1	3,2687	2,56421	Zfp352	5,1
Z00070787-1	3,3631	2,6655	EG668730	5,0
Z00009785-1	4,211	3,5136	Zfp296	5,0
Z00017558-1	2,9508	2,2535	Prkch	5,0
Z00022677-1	3,4246	2,72861	Ccl3	5,0
Z00072872-1	3,4432	2,7578	Bbc3	4,8
Z00064837-1	3,1918	2,5095	Glrx2	4,8
Z00057090-1	4,545	3,864	D030070L09Rik	4,8
Z00058806-1	4,8592	4,17981	EG434050	4,8
Z00059951-1	3,1707	2,4985	LOC434179	4,7
Z00021695-1	3,7753	3,1052	Slc27a2	4,7
Z00031419-1	3,3065	2,6373	C330013J21Rik	4,7
Z00016144-1	3,5578	2,8945	Arid4a	4,6
Z00052427-1	2,8283	2,1753	Stox1	4,5
Z00072669-1	3,4941	2,8428	LOC675962	4,5
Z00043376-1	4,6454	4,0058	Avpi1	4,4
Z00070167-1	3,6342	3,0073	1200016E24Rik	4,2
Z00057680-1	3,2262	2,6018	LOC381936	4,2
Z00030929-1	3,5962	2,9719	Rex2	4,2
Z00024913-1	3,9565	3,34101	Jam2	4,1
Z00010394-1	4,4632	3,85191	Tsen2	4,1
Z00050174-1	3,0756	2,46561	Tbc1d12	4,1

Z00071813-1	2,573	1,9662	LOC193079	4,0
Z00058060-1	3,4599	2,85471	Snip1	4,0
Z00012313-1	3,6579	3,0601	2410017P07Rik	4,0
Z00022630-1	3,0256	2,43211	Dub2	3,9
Z00034227-1	3,1045	2,53911	EG665756	3,7
Z00071997-1	2,9465	2,38351	LOC229571	3,7
Z00031864-1	2,949	2,38751	LOC545238	3,6
Z00061068-1	2,6306	2,071	4930488B22Rik	3,6
Z00072639-1	3,563	3,00441	5830484A20Rik	3,6
Z00025963-1	2,8235	2,2731	Serpina5	3,6
Z00004477-1	3,2955	2,749	Pthr1	3,5
Z00071412-1	3,2302	2,68481	Tdpoz5	3,5
Z00026921-1	2,9463	2,40431	EG620899	3,5
Z00030763-1	4,0011	3,4597	BC003993	3,5
Z00072792-1	3,8088	3,27261	LOC677603	3,4
Z00020938-1	3,672	3,1387	Scamp1	3,4
Z00024554-1	2,5009	1,9855	Chrnb4	3,3
Z00043435-1	3,9688	3,4572	Phf11	3,2
Z00038178-1	3,7741	3,26771	1700052K11Rik	3,2
Z00073573-1	2,9905	2,4888	D3Ertd731e	3,2
Z00020173-1	3,0655	2,5656	Adcy5	3,2
Z00071451-1	4,6067	4,11191	LOC673503	3,1
Z00070538-1	4,6619	4,16721	AI553587	3,1
Z00052608-1	3,5467	3,0532	Dcbld1	3,1
Z00063525-1	2,8581	2,37211	LOC623033	3,1
Z00023684-1	3,1333	2,6498	Bdnf	3,0
Z00035296-1	2,6883	2,20601	Hdc	3,0
Z00041617-1	2,9054	2,4233	4932442E05Rik	3,0
Z00064347-1	2,8518	2,37251	BC038328	3,0
